# Metabolomics Provides Quality Characterization of Commercial Gochujang (Fermented Pepper Paste)

**DOI:** 10.3390/molecules21070921

**Published:** 2016-07-15

**Authors:** Gyu Min Lee, Dong Ho Suh, Eun Sung Jung, Choong Hwan Lee

**Affiliations:** Department of Bioscience and Biotechnology, Konkuk University, Seoul 05029, Korea; alsl2930@naver.com (G.M.L.); sdh14031988@naver.com (D.H.S.); jes708@konkuk.ac.kr (E.S.J.)

**Keywords:** gochujang, quality characterization, metabolomics, GC-TOF-MS, UHPLC-LTQ-ESI-IT-MS/MS, antioxidant activity

## Abstract

To identify the major factors contributing to the quality of commercial gochujang (fermented red pepper paste), metabolites were profiled by mass spectrometry. In principal component analysis, cereal type (wheat, brown rice, and white rice) and species of hot pepper (*Capsicum* *annuum*, *C. annuum* cv. Chung-yang, and *C. frutescens*) affected clustering patterns. Relative amino acid and citric acid levels were significantly higher in wheat gochujang than in rice gochujang. Sucrose, linoleic acid, oleic acid, and lysophospholipid levels were high in brown-rice gochujang, whereas glucose, maltose, and γ-aminobutyric acid levels were high in white-rice gochujang. The relative capsaicinoid and luteolin derivative contents in gochujang were affected by the hot pepper species used. Gochujang containing *C. annuum* cv. Chung-yang and *C. frutescens* showed high capsaicinoid levels. The luteolin derivative level was high in gochujang containing *C. frutescens*. These metabolite variations in commercial gochujang may be related to different physicochemical phenotypes and antioxidant activity.

## 1. Introduction

Food is essential to sustain life and has undergone continuous development worldwide for a long time. As the industrialization of food products has progressed over the past few decades, the public interest in health and well-being has also increased [1]. To meet these demands, application of scientific knowledge to the food industry has become necessary to control quality and safety as well as to maintain the beneficial components in commercial food products [2]. However, various factors contribute to food quality, such as biological origin, ecological conditions, microbiome, and processing methods. Particularly, fermented foods contain more complex factors and mechanisms than other food types.

**Figure 1:**
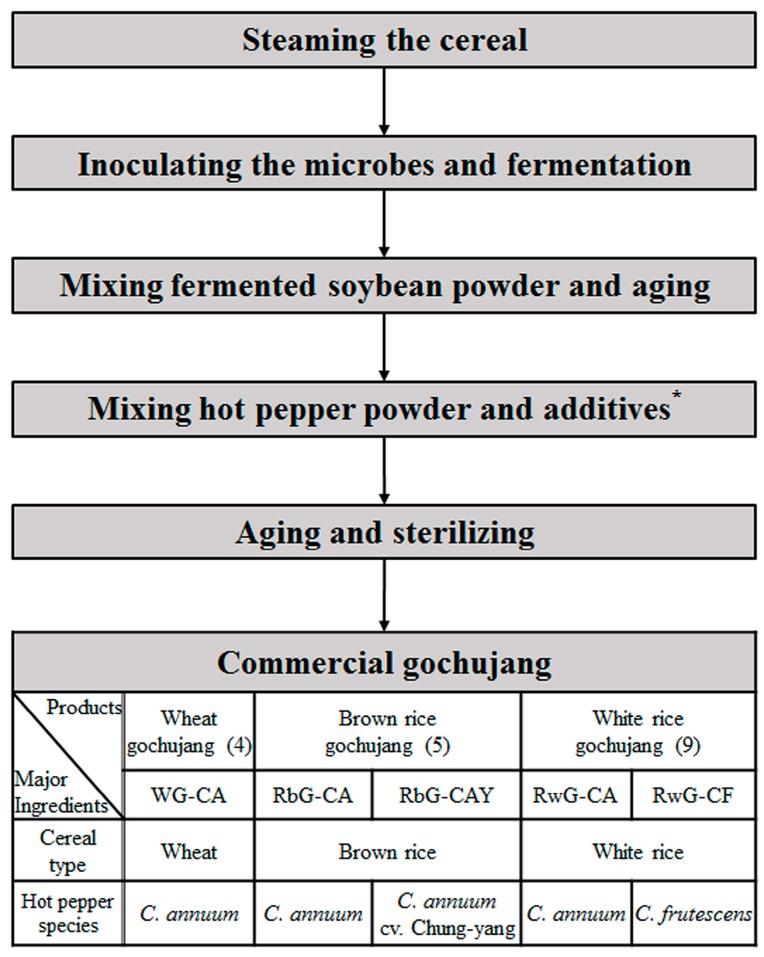
The schematic diagram of the general manufacturing process and the major raw ingredients for commercial gochujang. * Additives; salt, starch syrup, garlic, onion, and soy powder are typically on the ingredient label of the products.

Gochujang (fermented red pepper paste) is a traditional food that has unique properties, including spicy flavor, pungency, sweet taste, and distinctive texture. It is rich in nutrient components, such as essential amino acids, fatty acids, organic acids, and sugars, which are generated from raw materials during fermentation [3]. Furthermore, gochujang has various functional and biological activities, such as anti-obesity [4,5], anti-tumor [6], and anti-cancer [7] effects. The general manufacturing process for commercial gochujang is complex because of the combination of various raw materials and the diverse conditions required for production, which are summarized in Figure 1. Because of these complexities, it is difficult to control the quality of commercial gochujang. Thus, it is important to identify the major factors, as well as their mechanisms of action, affecting the quality of commercial gochujang.

Metabolomics have been developed in numerous fields, including pharmacology, toxicology, plant physiology, food science, and nutrition [8,9,10,11]. Metabolomics techniques are very important tools in food science in particular to ensure food safety, quality, and traceability [12]. For example, these methods can be applied to detect contaminants, pathogens, and toxins in food [13]; to evaluate the quality of fruits or beverages based on metabolite profiles [14]; and to monitor metabolic changes during storage and fermentation [15,16]. Metabolomics approaches are actively used to investigate fermented foods such as cheeses, wines, fermented soy products, and probiotics [17]. Metabolomics approaches have been used to evaluate gochujang to confirm the bioactivities of extracts or specific compounds and to investigate the bioconversion of compounds during fermentation [3,4,5,6,7]. However, few studies have examined differences in metabolites to characterize commercial gochujang.

In this study, the untargeted metabolite profiling of various commercial gochujang preparations was performed using GC-TOF-MS and ultrahigh performance liquid chromatography-linear trap quadrupole-ESI-ion trap-MS/MS (UHPLC-LTQ-ESI-IT-MS/MS) with multivariate statistical analysis to investigate the major factors contributing to the metabolite state and physicochemical quality of the final commercial form of gochujang. In addition, we propose specific metabolites as quality markers for commercial gochujang.

## 2. Results

### 2.1. Metabolite Profiling of Commercial Gochujang Samples

**Figure 2:**
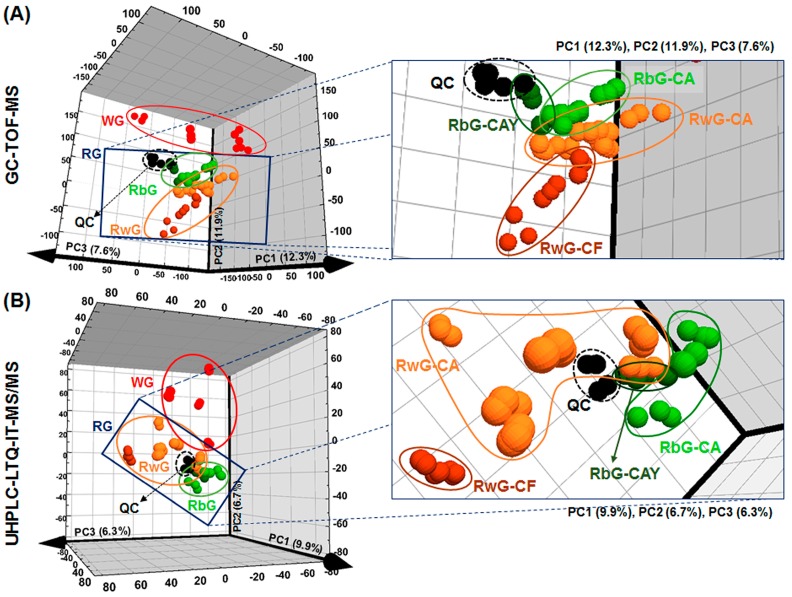
Principal component analysis (PCA) score plots of commercial gochujang derived from the GC-TOF-MS (**A**) and UHPLC-LTQ-IT-MS/MS (**B**) data sets; ● QC, quality control samples; ● WG, wheat gochujang; □ **RG**, rice gochujang (including RbG and RwG); ● RbG, brown rice gochujang; ● RwG, white rice gochujang; ● RbG-CA, brown rice gochujang containing *C. annuum*; ● RbG-CAY, brown rice gochujang containing *C. annuum* cv. Chung-yang; ● RwG-CA, white rice gochujang containing *C. annuum*; ● RwG-CF, white rice gochujang containing *C.* *frutescens*. Explained variances (R^2^X_(cum)_) and predictive abilities (Q^2^_(cum)_) of PCA models were 0.582 and 0.342 in (**A**) and 0.477 and 0.186 in (**B**), respectively.

To investigate the factors affecting the differences among the final products of a diverse group of gochujang samples (Figure 1 and Table 1) and to characterize the metabolites affecting the physicochemical quality of commercial gochujang, multivariate statistical analysis was performed using the GC-TOF-MS and UHPLC-LTQ-ESI-IT-MS/MS data sets (Figure 2). The variance of each PCs of the three-dimensional (3D) PCA score plots were PC1 (12.3%), PC2 (11.9%), and PC3 (7.6%) (Figure 2A) and PC1 (9.9%), PC2 (6.7%), and PC3 (6.3%) (Figure 2B). Although the total variances are low, the explained variances (R^2^) values of PCA models were over 0.4 (R^2^X_(cum)_ = 0.582 and 0.477), reasonably. And the distinct patterns for commercial gochujang are shown based on their raw materials in the 3D PCA score plots. As shown in Figure 2, the samples were mainly divided into two groups by cereal type, gochujang made with wheat (WG) and gochujang made with rice (RG). Within the cluster of RG, there was differentiation into groups of gochujang prepared using brown rice (RbG) and white rice (RwG). In the expanded figures, the two clusters of RbG and RwG were separated according to hot pepper species used to produce gochujang. The RbG cluster was separated into two groups based on the PCA score plot, including one with *C. annuum* (CA) and another mixed with *C. annuum* cv. Chung-yang (CAY). RwG was also divided into two clusters as follows: one prepared with CA and the other containing *C. frutescens* (CF). In summary, 18 commercial gochujang samples were separated and clustered based on cereal grain type (WG, RbG, and RwG) and secondarily on hot pepper species (RbG-CA, RbG-CAY, RwG-CA, and RwG-CF) in the PCA score plots (Figure 2).

### 2.2. Comparisons of Metabolites, Physicochemical Characteristics, and Antioxidant Activity According to Different Cereal Grains Used in Gochujang

**Figure 3:**
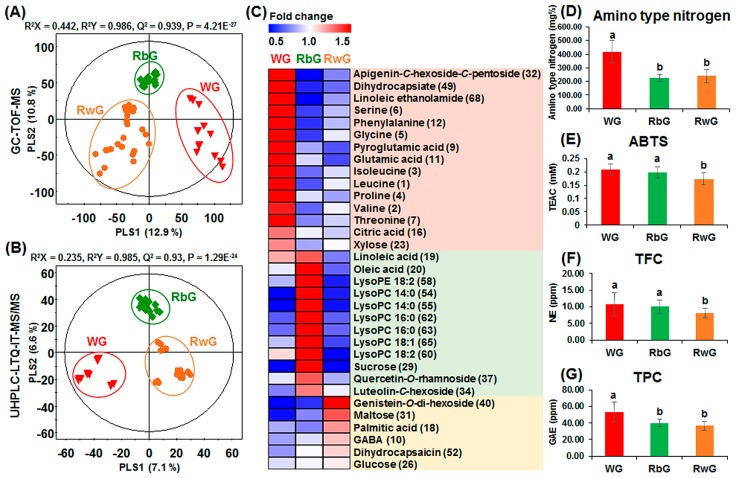
Partial least square-discriminate analysis (PLS-DA) score plots using the GC-TOF-MS (**A**); UHPLC-LTQ-ESI-IT-MS/MS (**B**) data sets, heat map of specific metabolites (**C**); amino type nitrogen contents (**D**); antioxidant activity tested by ABTS (**E**); total flavonoid contents (TFC) (**F**); and total polyphenolic contents (TPC) (**G**) according to the type of cereal grain used in commercial gochujang; ▼ WG, wheat gochujang; ♦ RbG, brown rice gochujang; ● RwG, white rice gochujang. The numbers in the heat map correspond to the compound numbers presented in Table S3. Different letters in the bar graph indicate significant difference by ANOVA followed by Duncan’s multiple-range test (*p*-value < 0.05).

To compare the differences in metabolites of gochujang according to the type of cereal grain used for preparation, PLS-DA was conducted using the GC-TOF-MS (Figure 3A) and UHPLC-LTQ-ESI-IT-MS/MS data (Figure 3B). The three groups of WG, RbG, and RwG were clearly separated from each other based on the PLS-DA score plots. The WG samples were separated from both the RbG and RwG groups by PLS1 (12.9% in Figure 3A and 7.1% in Figure 3B), while the RbG group was distinct from the WG and RwG groups according to PLS2 (10.8% in Figure 3A and 6.6% in Figure 3B). Significantly different variables were selected by PLS1 and PLS2 in the PLS-DA score plots based on variable importance in projection (VIP) values > 0.7 and *p*-values < 0.05. Next, primary and secondary metabolites (based on GC-MS and LC-MS/MS data sets, respectively) were tentatively identified by retention time, molecular weight, ultraviolet light lambda-max (λ_max_, nm), MS/MS fragment patterns, in-house library, library of National Institute of Standards and Technology (NIST MS Search program, version 2.0, Gaithersburg, MD, USA), standard compounds, and references (Tables S1 and S2).

Significantly different metabolites, including amino acids, organic acids, fatty acids, sugars and sugar alcohols, flavonoids, capsaicinoids and capsinoids, and lipids, according to the type of cereal grains used in gochujang were identified, and their relative contents were determined (Table S3). Among them, metabolites with significantly distinct patterns (*p*-value < 0.05) such as unique high or low contents in specific gochujang groups (WG, RbG, and RwG) were visualized with a heat map (Figure 3C). The heat map was prepared using the fold-change values normalized to the average relative contents of each metabolite in all gochujang samples. The levels of most amino acids [leucine (**1**), valine (**2**), isoleucine (**3**), proline (**4**), glycine (**5**), serine (**6**), threonine (**7**), pyroglutamic acid (**9**), glutamic acid (**11**), and phenylalanine (**12**)], citric acid (**16**), xylose (**23**), and 3 secondary metabolites [apigenin-*C*-hexoside-*C*-pentoside (**32**), dihydrocapsiate (**49**), and linoleic ethanolamide (**68**)] were distinctively higher in the WG group than in the other samples. Lipid-related molecules such as fatty acids [linoleic acid (**19**) and oleic acid (**20**)] and lysophospholipids [lysoPC 14:0 (**55**), lysoPC 18:2 (**60**), lysoPC 16:0 (**63**), lysoPC 18:1 (**65**), and lysoPE 18:2 (**58**)], sucrose (**29**), and two other secondary metabolites including luteolin-*C*-hexoside (**34**) and quercetin-*O*-rhamnoside (**37**) were present at significantly higher levels in the RbG group than in the other samples. For RwG, some sugars such as glucose (**26**) and maltose (**31**) were present at higher levels than in the other samples. In addition, the relative contents of two other primary metabolites (γ-aminobutyric acid (GABA) (**10**) and palmitic acid (**18**)) and two secondary metabolites (genistein-*O*-dihexoside (**40**) and dihydrocapsaicin (**52**)) were also high in the RwG group.

The results for antioxidant activity, TPC, TFC, and physicochemical characteristic assays (amino type nitrogen, acidity, pH, salinity, and reducing sugar content) for the commercial gochujang samples are presented according to the type of cereal grain in Figure 3 and Figure S1. The values for amino type nitrogen and TPC were the highest in WG (Figure 3D,G), as were the values for acidity and salinity (Figure S1). Antioxidant activity, TFC, and pH were higher in WG and RbG than in RwG (Figure 3E,F and Figure S1), whereas reducing sugar content was significantly higher in WG and RwG than in RbG (Figure S1).

### 2.3. Metabolite Variations and Antioxidant Activity According to Species of Hot Pepper Used in Gochujang

**Figure 4:**
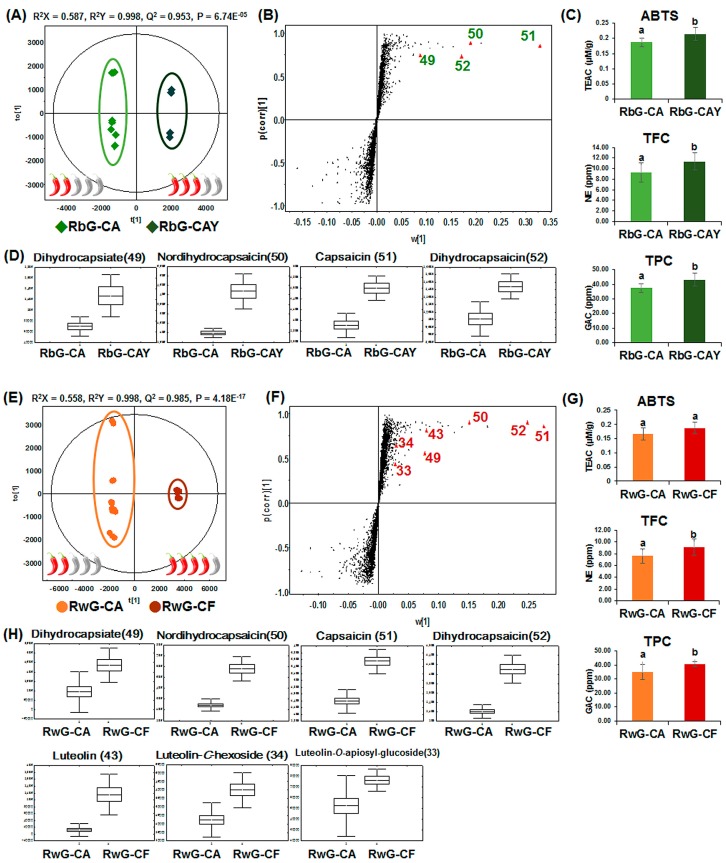
Orthogonal partial least squares discriminant analysis (OPLS-DA) score plots (**A**,**E**), loading S-plots (**B**,**F**), box-and-whisker plots (**D**,**H**) of significantly different metabolites derived from the UHPLC-LTQ-IT-MS/MS data set, and the results for ABTS, TFC, and TPC (**C**,**G**) for brown rice (**A**–**D**) and white rice (**E**–**H**) commercial gochujang according to species of hot pepper present; ♦ RbG-CA, brown rice gochujang containing *C. annuum*; 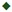 RbG-CAY, brown rice gochujang containing *C. annuum* cv. Chung-yang; ● RwG-CA, white rice gochujang containing *C. annuum*; ● RwG-CF, white rice gochujang containing *C. frutescens.* Relative pungency levels of hot peppers classified by Scoville heat units (SHU); 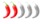 mildly pungent (700–3000 SHU), 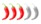 moderately pungent (3000–25,000 SHU), and 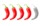 highly pungent (25,000–70,000 SHU) [18]. The metabolite numbers are identical to those in Table S2. Different letters in the bar graph indicate significant differences by ANOVA followed by Duncan’s multiple-range test (*p*-value < 0.05). The red pepper diagram is the relative pungency levels of hot pepper.

According to the PCA models, which showed different patterns according to minor factors and the ingredient labels on the products, the gochujang samples were categorized based on the different species of hot pepper present, including RbG-CA, RbG-CAY, RwG-CA, and RwG-CF (Table 1 and Figure 2). To investigate the metabolites contributing to differentiation according to species of hot pepper in RbG or RwG, OPLS-DA was conducted using the MS spectrum data set from UHPLC-LTQ-ESI-IT-MS/MS (Figure 4A,D), and variables were selected using VIP values (>1) and *p*-values (<0.05). These metabolites are presented in Table S2. Among them, specific pepper-related variables were highlighted in the loading S-plots (Figure 4B,F), and their relative contents were visualized using box-and-whisker plots (Figure 4D,H). The antioxidant activity, TFC, and TPC results are presented in Figure 4C,G. In a comparison of RbG-CA and RbG-CAY (Figure 4B–D), a total of 19 metabolites were identified as differential metabolites (Table S2), among which the relative levels of capsaicinoids and capsinoids, including nordihydrocapsaicin (**50**), capsaicin (**51**), dihydrocapsaicin (**52**), and dihydrocapsiate (**49**), were significantly higher in RbG-CAY than in RbG-CA. These patterns were similar to the results for ABTS, TFC, and TPC, which were significantly higher in RbG-CAY than in RbG-CA. In a comparison of RwG-CA and RwG-CF (Figure 4F–H), a total of 30 metabolites were identified (Table S2), among which the relative levels of luteolin-*O*-apiosyl-glucoside (**33**), luteolin-*C*-hexoside (**34**), luteolin (**43**), dihydrocapsiate (**49**), nordihydrocapsaicin (**50**), capsaicin (**51**), and dihydrocapsaicin (**52**) were higher in RwG-CF than in RwG-CA (Figure 4H).

Specifically, the levels of luteolin and luteolin derivatives were significantly different only in the comparison between RwG-CA and RwG-CF. These metabolite variations were similar to the patterns for ABTS, TFC, and TPC, which showed higher levels in RwG-CF than in RwG-CA.

## 3. Discussion

The quality of fermented foods is affected by numerous factors such as the type of raw materials, diversity of microbes used in fermentation, and other fermentation conditions [16,17,19]. The quality of gochujang, a fermented red pepper paste, is affected by diverse factors derived from the complex manufacturing process, including the conditions used for the steaming, fermentation, and aging procedures; numerous raw materials used such as cereal grains, fermented soy powder, hot pepper powder, salt, and other additives; and their combinations (Figure 1). In this study, we conducted the untargeted metabolite profiling with several physicochemical assays on commercial gochujang samples to identify the factors important for the quality characterization of commercial gochujang. Based on the results of metabolite profiling, clustering patterns were determined using PCA models, with different cereal grains as main factors and the species of hot pepper as minor factors (Figure 2).

In WG, most amino acids were present in relatively high levels (Figure 3C). In general, amino acids play important roles in the aroma, flavor, and nutritional quality of food products. Aspartic acid (**8**), glutamic acid (**11**), and phenylalanine (**12**) are related to the umami flavor [20]; proline (**4**) and threonine (**7**) contribute to the sweet flavor; and leucine (**1**), valine (**2**), and isoleucine (**3**) are related to the bitter flavor [17,19]. The other amino acids such as glycine (**5**), serine (**6**), and pyroglutamic acid (**9**) were also high in WG. Aspartic acid (**8**) and glutamic acid (**11**) are acidic amino acids and likely contributed to the high acidity of WG (Figure S1). Amino acids have been shown to contribute to antioxidant activity [21], and the high levels of amino acids in WG might have affected the patterns of ABTS and amino type nitrogen content (Figure 3D,E). Furthermore, these specific metabolite states may be related to the natural characteristics of the grain storage proteins known as wheat prolamins [22]. These proteins are generally present in cereal grains containing starch and some lipids, and the proportion of these proteins in wheat grains is higher than in rice seeds [22,23]. These common structural features related to specific amino acid residues lead to high proportions of glutamine, proline, and other amino acids (e.g., glycine, methionine, and phenylalanine) [22]. In addition, the levels of organic acids such as fumaric acid (**14**), malic acid (**15**), citric acid (**16**), and gluconic acid (**17**) were also high in WG (Table S3 and Figure 3). Citric acid is widely used as an additive in the food industry and contributes to a sour taste along with other organic acids [24]. Based on our results, the general properties of wheat grains affected taste-related components such as amino acids and organic acids and the quality characteristics of the final product, WG.

In the RG (RbG and RwG), lipid-related components and some sugars were present at significantly higher levels than in WG (Table S3 and Figure 3). The average values of ABTS, TFC, and TPC were higher in RbG than in RwG. First, brown rice (*Oryzae sativa*) possesses bran layers containing abundant phenolic compounds and other nutritional components such as essential fatty acids and various lipids [25]. In RbG, the relative levels of most fatty acids; lipids such as linoleic acid (**19**), oleic acid (**20**), lysoPC 14:0 (**55**), lysoPE 18:2 (**58**), lysoPC 18:2 (**60**), lysoPC 16:0 (**63**), and lysoPC 18:1 (**65**); and sucrose (**29**) were higher than in the other gochujang samples. The high levels of these specific metabolites in RbG were attributed to the nutritional properties of brown rice. Rice bran phospholipids consist of palmitic (16:0), oleic (18:1), and linoleic (18:2) acids, as well as lysoPC 16:0, lysoPC 18:1, and lysoPC 18:2, in the outer section of the rice grain [26,27]. Ninety percent of the free sugar in rice grain is sucrose, and more than 60% of this sugar is present in the outer layer of the rice grain. Thus, the sucrose content of brown rice is generally higher than that of milled (white) rice [28]. Furthermore, various health-promoting effects of phospholipids such as lowering cholesterol and cardiovascular risk, inhibiting metastasis, regulating inflammatory reactions, and improving immunological functions have been reported [27]. Therefore, brown rice may be useful for producing fermented foods that contain more lipids than products made using wheat or white rice as raw materials. Second, white rice is composed of 80%–90% starch and is milled more than brown rice. In RwG, the relative levels of glucose (**26**) and maltose (**31**) were higher than in RbG and WG (Table S3 and Figure 3). The levels of these metabolites in RwG were affected by the properties of the white rice grains. The starch level in the endosperm is higher than on the grain surface [27,29] and is composed of amylose and amylopectin. During digestion or fermentation, glucose and maltose are produced through hydrolysis [29]. According to Shu et al., the level of amylose-lipid complexes is lower in white rice than in brown rice. A high ratio of amylose-lipid complexes reduces the decomposition sensitivity of rice starch by enzymes such as α-amylase [30]. Amylose hydrolysis of white rice occurs at a faster rate than for brown rice. Thus, fermented foods prepared with white rice may be sweeter than those prepared with brown rice without any other additives.

As shown in Figure 4, the significantly different metabolite levels and the results of three assays were visualized according to the hot pepper species used in gochujang. The values for antioxidant activity, TFC, and TPC were higher in gochujang containing CAY or CF (RbG-CAY and RwG-CF) than in gochujang containing CA (RbG-CA and RwG-CA) (Figure 4C,G). These patterns closely corresponded to the variation in capsaicinoids and capsinoids such as dihydrocapsiate (**49**), nordihydrocapsaicin (**50**), capsaicin (**51**), and dihydrocapsaicin (**52**), which are known to have antioxidant activity (Figure 4D,H) [31]. Most capsaicinoids are pungent, and the pungency level of each compound is described in Scoville heat units (SHU). The reported pungency levels of capsaicin (**51**) and dihydrocapsaicin (**52**) are 1.5 × 10^7^ SHU, whereas that of nordihydrocapsaicin (**50**) is 9.1 × 10^6^ SHU. Capsinoids, which are capsaicinoid-like substances, such as capsiate and dihydrocapsiate (**49**) are not pungent [32]. These pungency-related metabolites present at high levels in RbG-CAY and RwG-CF resulted from the general characteristics of the hot peppers. According to Choi et al. CAY pepper was found to contain the highest levels of total capsaicinoids among 11 Korean red peppers [33]. The CF pepper, known as “Bird chili” or “Thai chili”, is widely used in Thailand. This hot pepper has a tiny cone shape and is highly pungent, with SHU values of 5 × 10^4^–1 × 10^5^ SHU [32]. In addition, some flavonoids, including luteolin-*O*-apiosyl-glucoside (**33**), luteolin-C-hexoside (**34**), and luteolin (**43**), were selected as metabolites to differentiate between RwG-CA and RwG-CF (Figure 4F,H), which showed similar patterns for TFC (Figure 4G). The levels of these metabolites were higher in RwG-CF than in RwG-CA. Our findings are consistent with those of Wahyuni et al., who evaluated the differences in 32 *Capsicum* spp. pepper fruits in terms of flavonoids such as glycosyl derivatives of quercetin, luteolin, and apigenin and their relative levels [34]. The relative levels of luteolin derivatives are high in *C. frutescens*, which is the species used to prepare RwG-CF; other samples used different species such as *C. annuum*. Thus, our data suggest that CAY and CF peppers can be used to control the level of pungency and alter the flavonoid contents in gochujang.

## 4. Materials and Methods

### 4.1. Chemicals and Reagents

Methanol, acetonitrile, and water were purchased from Fisher Scientific (Pittsburgh, PA, USA). Formic acid, methoxyamine hydrochloride, pyridine, *N*-methyl-*N*-(trimethylsilyl)trifluoroacetamide (MSTFA), diethylene glycol, gallic acid, naringin, 6-hydroxy-2,5,7,8-tetramethylchroman-2-carboxylic acid (Trolox), hydrochloric acid, potassium persulfate, 2,2′-azinobis(3-ethylbenzothiazoline-6-sulfonic acid) diammonium salt (ABTS), Folin-Ciocalteu′s phenol reagent, 37% formaldehyde solution, sodium hydroxide solution, dinitrosalicylic acid (DNS), formononetin, norvaline, and other analytical standard compounds were purchased from Sigma-Aldrich (St. Louis, MO, USA).

### 4.2. Sample Preparation

#### 4.2.1. Sample Information

Eighteen commercial gochujang samples were purchased from a local market (Seoul, Korea) and were stored at 4 °C until analysis. Based on the ingredient labels of the gochujang samples, the raw material information is summarized in Table 1. In addition, the general manufacturing process for commercial gochujang with the raw material information for the samples used in this study are schematized in Figure 1. As shown in Figure 1, diverse raw materials were used to produce the commercial gochujang samples using a highly complex process. After the cereal grains were steamed, a pure strain of *Aspergillus* or *Bacillus* spp. was inoculated into the steamed cereals and fermented to form a *koji*. Next, the *koji* was mixed with fermented soybean powder and aged for approximately 20 days. Hot pepper powder and other additives such as salt, starch syrup, garlic, and onion were mixed, and the product was aged and sterilized. Finally, the commercial gochujang was packed. In this study, a total of 18 commercial gochujang samples prepared from three types of cereal grains, including wheat, brown rice, and white rice, and different species of hot pepper powder, including *Capsicum annuum* (CA), *C. annuum* cv. Chung-yang (CAY), and *Caspsicum frutescens* (CF), were used; the combinations of the major raw materials are shown in Figure 1. The general relative pungency levels of hot pepper fruits [18] are described using red pepper diagrams with other information for each sample in Table 1.

#### 4.2.2. Sample Extraction and Derivatization for Metabolite Profiling

Each commercial gochujang sample (300 mg) was extracted using 80% methanol (800 µL) with formononetin (10 µL, 600 ppm) as an internal standard. The samples were sonicated for 15 min and homogenized using a Retsch MM400 Mixer Mill (Retsch GmbH & Co., Haan, Germany) at 30 Hz for 3 min. After centrifugation for 5 min at 11,000× *g* and 4 °C, the supernatants were collected, and the residues were extracted again using same procedure. The supernatants were filtered through a 0.2-μm polytetrafluoroethylene (PTFE) syringe filter and completely concentrated using a speed-vacuum concentrator (Biotron, Seoul, Korea). Dried extracts were re-suspended in 80% methanol to a final concentration of 100,000 ppm (100 mg/mL) and filtered. For the UHPLC-LTQ-ESI-IT-MS/MS analysis, the resolved extracts were diluted to 50,000 ppm (50 mg/mL). For the GC-TOF-MS analysis, 5 µL of norvaline (20 mg/mL) was added to 50 μL (100 mg/mL) of the extracts as an internal standard and concentrated again. Derivatized extracts were obtained by oximation and silylation. First, dried extracts were oximated with 100 µL of methoxyamine hydrochloride in pyridine (20 mg/mL) at 30 °C for 90 min. Next, the extracts were silylated with 100 μL of MSTFA at 37 °C for 30 min. The final concentration of the derivatized extracts was set to 25,000 ppm (25 mg/mL). To evaluate the technical variables including extraction, sample analysis, and data processing, quality control (QC) samples were also prepared by pooling 10 µL of each sample for both GC-MS and LC-MS analyses. The extracts were prepared at a final concentration of 10,000 ppm (10 mg/mL) for the antioxidant activity tests.

### 4.3. GC-TOF-MS Analysis

GC-TOF-MS analysis was performed using an Agilent 7890A GC system coupled with an Agilent 7693 autosampler (Agilent, Santa Clara, CA, USA) equipped with a Pegasus HT TOF-MS (LECO Corp., St. Joseph, MI, USA) system. An Rtx-5MS capillary column (30 m × 0.25 mm i.d.; 0.25 μm particle size; Restek Corp., Bellefonte, PA, USA) was used with helium gas and a flow rate of 1.5 mL/min. The front inlet and transfer line temperatures were 250 °C and 240 °C, respectively. The starting oven temperature was maintained for 2 min at 75 °C, increased to 300 °C for 15 min at a rate of 15 °C/min, and then maintained at 300 °C for 3 min. The ion source temperature and electron energy were set to 230 °C and −70 eV, respectively. The detector voltage was 1600 V, and the scanning mass range was 45–800 *m*/*z*. One microliter of derivatized sample was injected into the GC with a split ratio of 10:1. Following a randomized injection order, three analytical replications were analyzed for each gochujang sample. To evaluate the technical variables, we analyzed spiked QC samples for each 15 samples analyzed.

### 4.4. UHPLC-LTQ-ESI-IT-MS/MS Analysis

To analyze the gochujang extracts, UHPLC-LTQ-ESI-IT-MS/MS was used. The LTQ XL linear ion trap mass spectrometer is consist of an electrospray interface (Thermo Fisher Scientific, Inc., Waltham, MA, USA) coupled to a DIONEX UltiMate 3000 RS Pump, RS Autosampler, RS Column Compartment, and RS Diode Array Detector (Dionex Corporation, Sunnyvale, CA, USA). Ten microliters of each sample was injected and separated using a Thermo Scientific Syncronis C18 UHPLC column (100 mm × 2.1 mm i.d.; 1.7 μm particle size). The mobile phase consisted of A (0.1% formic acid in water, *v*/*v*) and B (0.1% formic acid in acetonitrile, *v*/*v*). The flow rate was 0.3 mL/min. The initial condition was 10% of solvent B for 1 min. Next, the gradient was linearly increased to 100% of solvent B over 14 min and maintained for 3 min. After then, the gradient was reduced to 10% for 1 min, followed by re-equilibration of the column to the initial conditions for 3 min. The photodiode array was set to 200–600 nm for detection and managed by a 3D field. Ion trapping was performed in positive, negative, and full-scan ion modes within a range of 150–1000 *m*/*z*. The operating parameters were set as follows: source voltage, ±5 kV; capillary voltage, 39 V; and capillary temperature, 275 °C. After the full-scan MS analysis, tandem MS (MS/MS) analysis was carried out to collect MS^n^ information by scan-type turbo data-dependent scanning (DDS), under the same conditions in both positive and negative ion modes. Among ions separated by mass-to-charge (*m*/*z*) ratio in the first stage of mass spectrometry (MS^1^), the first and second most intense ion (precursor ion) are selected and subjected to collision-induced dissociation (CID) to create fragment ions. The product ions are separated and detected in the MS^2^, and so on. The normalized collision energy was set to 35% and the dwell times in the trap were set to 25 ms. Three analytical replications were analyzed for each gochujang sample. To evaluate the technical variables, we spiked QC samples for each 15 samples analyzed.

### 4.5. Data Processing and Multivariate Analysis

After the GC-TOF-MS and UHPLC-LTQ-ESI-IT-MS/MS analyses, raw data files were converted into a computable document format (*.cdf) using LECO Chroma TOF software (Version 4.4, LECO Corp.) and using the thermo file converter program in Thermo Xcalibur software (version 2.1, Thermo Fisher Scientific). The acquired NetCDF format (*.cdf) files were processed to determine retention time, baseline correction, peak detection, and alignment using the MetAlign software package [35]. After alignment, the resulting data file (*.csv) including the corrected peak retention times, peak areas, and corresponding mass (*m*/*z*) data was exported to Microsoft Excel (Microsoft, Redmond, WA, USA) for further analysis. Multivariate statistical analysis using SIMCA-P+ 12.0 software (version 12.0, Umerics, Umea, Sweden) was performed to compare metabolite differences between commercial gochujang samples by unsupervised principal component analysis (PCA), supervised partial least squares discriminant analysis (PLS-DA), and orthogonal PLS-DA (OPLS-DA). Significant differences (*p*-value < 0.05) between selected metabolites, their relative contents, and the values from the physicochemical characteristics assays were evaluated by one-way analysis of variance using SPSS 18.0 (SPSS Inc., Chicago, IL, USA). The differences between metabolites were visualized by heat map analysis using MEV software [36].

### 4.6. Determination of Antioxidant Activity (by ABTS), Total Polyphenol Content (TPC), and Total Flavonoid Content (TFC)

Three assays—ABTS, TPC, and TFC—were conducted using procedures described by Jung et al. (2013) with slight modifications [10]. For the ABTS test, a dark-blue-colored stock solution was prepared by dissolving 7 mM ABTS in a 2.45 mM potassium persulfate solution, after which it was maintained in the dark for 24 h. The solution was diluted to reach an absorbance 0.7 ± 0.03 at 750 nm using a microplate reader (Spectronic Genesys 6, Thermo Electron, Madison, WI, USA). Then, 10 μL of the extracted sample was added to the diluted ABTS solution (190 μL) in a 96-well plate. After 6 min in dark, absorbance was measured at 750 nm using a microplate reader. The result was presented as Trolox equivalent activity concentration (TEAC), with the standard solution concentration curve ranging from 0.015 mM to 1.000 mM.

For the analysis of total polyphenolic content (TPC), the Folin-Ciocalteu colorimetric method was used. In brief, 0.2 N Folin-Ciocalteu’s phenol reagent (100 μL) was added to 20 μL of each sample in a 96-well plate, followed by incubation for 5 min in the dark. Then, a 7.5% sodium carbonate solution (80 μL) was added. The mixture was allowed to react for 60 min, and the absorbance was measured at 750 nm using a microplate reader. The result was calculated as gallic acid equivalent concentration (GAE), and the standard solution concentration curve ranged from 3.906 ppm to 500 ppm.

The total flavonoid content (TFC) was determined by measuring the absorbance of the reacted samples placed in 96-well plates. Briefly, 90% diethylene glycol (180 μL), 1 N NaOH (20 μL), and each extracted sample (20 μL) were mixed and reacted in the dark. After 60 min, the absorbance was recorded at 405 nm using a microplate reader. The result was expressed as naringin equivalent concentration (NE). The range of the standard solution concentration curve was from 1.562 ppm to 200 ppm. All experiments were performed in triplicate.

### 4.7. Analysis of Salinity, pH, Total Acidity, Amino Type Nitrogen, and Reducing Sugar Contents

To evaluate physicochemical characteristics, each gochujang sample (10 g) was mixed with 100 mL of distilled water, homogenized on a vortexer for 2 min, and sonicated for 1 min. After the mixed solutions were centrifuged for 10 min at 4 °C and 4000× *g*, the supernatants were collected and divided into three 100-mL beakers (20 mL each) to determine pH, total acidity, and amino type nitrogen.

The pH was determined using a pH meter (Orion 3 Star pH benchtop, Thermo Fisher Scientific, Inc.). Total acid contents and amino type nitrogen were calculated by the formol titration method as described by Kim et al. with some modifications [37]. Total acidity was calculated by titration of the sample solution to pH 8.4 with a 0.1 N sodium hydroxide solution. After the titration, the consumed amounts of the sodium hydroxide solution (*V*_1_) was converted into percent lactic acid using the following formula: total acidity (%) = ((0.009 × *V*_1_ × *D* × *F*)/*S*) × 100, where 0.009 is the conversion factor for lactic acid. *V*_1_ is the first consumption volume of the sodium hydroxide solution (mL), and *D*, *F*, and *S* are the dilution rate (10), factor of the 0.1 N sodium hydroxide solution (1.0028), and the sample amount (10 g), respectively.

To determine the amino type nitrogen contents, 20 mL of neutralized formaldehyde (37%, pH 8.4) was added to the above titrated sample solutions. After 1 min, the sample solutions were re-titrated to pH 8.4 with a 0.1 N sodium hydroxide solution (*V*_2_). The amount of the 0.1 N sodium hydroxide solution consumed (*V*_2_) was used to calculate the amino type nitrogen value. The milligram percentage of amino type nitrogen was expressed using the following formula: amino type nitrogen (mg%) = ((*V*_2_ × 1.4 × *D* × *F*)/*S*) × 100, where *V*_2_ is the second consumption volume of the sodium hydroxide solution (mL) and 1.4 is the nitrogen equivalent amount in 1 mL of the 0.1 N sodium hydroxide solution. Sample salinity (200 µL per each) was determined using a portable refractometer for salt measurements (HANNA Instruments, Inc., Padova, Italy).

The reducing sugar contents of each gochujang sample were estimated using the dinitrosalicylic acid (DNS) method with some modifications; glucose was used as a standard [38]. To detect reducing sugars, a mixture of DNS solution (400 µL) and the prepared samples (200 µL) was boiled for 5 min and then placed on ice for 10 min. Next, the mixtures were diluted with distilled water to a measureable concentration range. The absorbance of the diluted mixtures was measured using a spectrophotometer (Genesys 6, Thermo Electron Co., Waltham, MA, USA) at 550 nm. All assays were conducted in duplicate.

## 5. Conclusions

Through metabolite profiling, we found that metabolites related to cereal grain types and hot pepper species were major factors that distinguished different commercial gochujang samples. Characteristic metabolites such as amino acids, citric acid, fatty acids, and some sugars showed significant patterns depending on cereal grain type. The relative contents of capsaicinoids, luteolin, and luteolin derivatives were affected by the species of hot pepper used to prepare the gochujang. These metabolites may be useful as quality markers for commercial gochujang using a metabolomics approach. Furthermore, metabolomics approaches may also be suitable for characterization based on differential factors in other fermented foods.

## Figures and Tables

**Table 1:** Information for commercial gochujang samples.

Sample No.	Type of Cereal	Species of Hot Pepper ^a^	Pungency ^b^	Abbreviation ^c^
1	Wheat gochujang (WG)	*Capsicum annuum*	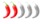	WG-CA
2				
3				
4				
5	Brown rice gochujang (RbG)	*Capsicum annuum*	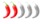	RbG-CA
6				
7				
8		*Capsicum annuum* cv. Chung-yang	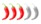	RbG-CAY
9				
10	White rice gochujang (RwG)	*Capsicum annuum*	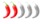	RwG-CA
11				
12				
13				
14				
15				
16		*Capsicum frutescens*	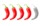	RwG-CF
17				
18				

^a^ Main species of pepper powder were indicated by the ingredients label of products. ^b^ Relative pungency levels of hot peppers classified by Scoville heat units (SHU); 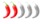 mildly pungent (700–3000 SHU); 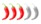 moderately pungent (3000–25,000 SHU); and 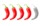 highly pungent (25,000–70,000 SHU) [18]. ^c^ Abbreviation of total samples used in the text; WG, wheat gochujang; RG, rice gochujang; RbG, brown rice gochujang; RwG, white rice gochujang; RbG-CA, brown rice gochujang containing *C. annuum*; RbG-CAY, brown rice gochujang containing *C. annuum* cv. Chung-yang; RwG-CA, white rice gochujang containing *C. annuum*; RwG-CF, white rice gochujang containing *C. frutescens*.

## Supplementary Materials

Supplementary materials can be accessed at: http://www.mdpi.com/1420-3049/21/7/921/s1.

## Abbreviations

The following abbreviations are used in this manuscript:

PCA	principal component analysis
PLS-DA	partial least squares discriminant analysis
OPLS-DA	orthogonal partial least squares discriminant analysis
VIP	variable importance in projection
MS	mass spectrometry
GC-TOF-MS	gas chromatography-time of flight-mass spectrometry
UHPLC-LTQ-ESI-IT-MS/MS	ultrahigh performance liquid chromatography-linear trap quadrupole-electrospray ionization-ion trap-MS/MS
MSTFA	*N*-methyl-*N*-(trimethylsilyl) trifluoroacetamide
DNS	dinitrosalicylic acid
Trolox	6-hydroxy-2,5,7,8-tetramethylchroman-2-carboxylic acid
ABTS	2,2′-azinobis(3-ethylbenzothiazoline-6-sulfonic acid)diammonium salt
TFC	total flavonoid content
TPC	total polyphenolic content
TEAC	Trolox equivalent activity concentration
NE	naringin equivalent
GAE	gallic acid equivalent
CA	*C.* *annuum*
CAY	*C. annuum* cv. Chung-yang
CF	*C. frutescens*
WG	wheat gochujang
RG	rice gochujang
RbG	brown rice gochujang
RwG	white rice gochujang
RbG-CA	brown rice gochujang containing *C.* *annuum*
RbG-CAY	brown rice gochujang containing *C.* *annuum* cv. Chung-yang
RwG-CA	white rice gochujang containing *C.* *annuum*
RwG-CF	white rice gochujang containing *C. frutescens*
