# Supplementary material for: Metabolomics Provides Quality Characterization of Commercial Gochujang (Fermented Pepper Paste)
Source: Molecules. 2016 Jul 15;21(7):921. doi: 10.3390/molecules21070921 (PMC6273659; doi:10.3390/molecules21070921)
Supplement: Supplementary file 1 [file molecules-21-00921-s001.pdf]

Supplementary Materials: Metabolomics Reveals Quality Characterization of Commercial Gochujang (Fermented Pepper Paste)

Gyu Min Lee, Dong Ho Suh, Eun Sung Jung and Choong Hwan Lee

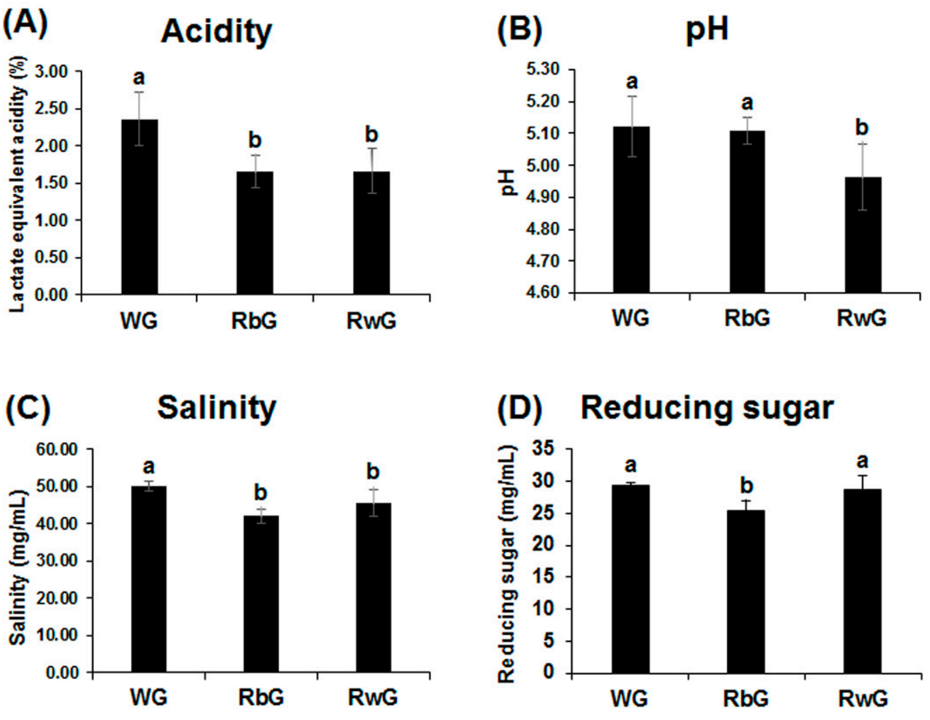

Figure S1. Graphs of the results for acidity (**A**), pH (**B**), salinity (**C**), and reducing sugar content (**D**) for physicochemical characteristics of commercial gochujang samples according to the different cereal grains used. All assays were conducted in duplicate, and values are the average of each group of gochujang (WG, wheat gochujang; RbG, brown rice gochujang; RwG, white rice gochujang). Different letters over the bars indicate that values were statistically significant by analysis of variance (ANOVA) followed by Duncan's multiple-range test (p-value < 0.05).

Table S1. List of significantly different metabolites according to type of cereal grains used in commercial gochujang based on GC-TOF-MS data.

No.	Tentative Metabolites [a]	VIP 1 [a]	VIP 2 [a]	t_R [b] (min)	MS Fragment Ion (*m*/*z*)	Identified Ion [c] (*m*/*z*)	TMS [d]	ID [e]
					Amino acids			
1	Leucine	1.16	1.49	5.91	86, 75, 73, 103, 61, 146, 188	86	1	MS
2	Valine	0.95	1.29	6.56	144, 73, 147, 45, 218, 100, 59, 133, 103	218	2	MS
3	Isoleucine	1.19	1.28	7.33	73, 158, 75, 117, 147, 45, 218, 100, 133, 130	158	2	MS
4	Proline	1.73	1.36	7.38	142, 73, 45, 143, 59, 216, 100, 133	142	2	MS
5	Glycine	1.26	1.61	7.46	73, 174, 147, 86, 45, 100, 59, 175, 248, 133, 75,	174	3	MS
6	Serine	1.55	1.56	7.96	73, 100, 188, 147, 45, 204, 59, 114, 133, 218, 262	204	3	MS
7	Threonine	1.22	1.21	8.21	73, 57, 117, 101, 219, 218, 45, 147, 100, 129, 133, 291	219	3	MS
8	Aspartic acid	0.08	1.25	8.51	73, 160, 75, 130, 117, 116, 45, 147, 100, 245, 234, 202	160	2	MS
9	Pyroglutamic acid	2.55	2.02	9.40	156, 73, 147, 45, 230, 258	156	2	MS
10	GABA	1.37	1.14	9.42	73, 156, 174, 147, 45, 86, 59, 304, 216, 246	174	3	MS
11	Glutamic acid	1.78	1.38	10.13	73, 246, 75, 128, 147, 84, 156, 45, 56, 230, 100	246	3	MS
12	Phenylalanine	1.42	1.45	10.23	73, 218, 192, 100, 45, 147, 59, 133, 266,	218	2	MS
					Organic acids			
13	Propanoic acid	1.08	1.90	7.69	73, 147, 189, 133, 45, 102, 103, 117, 292, 205	189	3	MS
14	Fumaric acid	2.25	1.90	7.78	147, 73, 245, 75, 99, 45, 241, 133, 113,	245	2	MS
15	Malic acid	2.23	1.72	9.08	73, 147, 75, 45, 133, 55, 233, 101, 189, 245, 117	233	3	MS
16	Citric acid	1.34	1.04	11.66	73, 147, 273, 45, 211, 133, 183, 347, 375, 257	273	4	MS
17	Gluconic acid	0.51	1.34	12.96	73, 147, 217, 103, 75, 205, 45, 129, 117, 333, 292, 133	333	6	MS
					Fatty acids			
18	Palmitic acid	1.07	1.23	13.10	73, 75, 117, 129, 147, 132, 217, 45, 55, 131, 205	129	1	MS
19	Linoleic acid	0.92	1.15	14.06	73, 75, 67, 81, 129, 55, 117, 80 95, 337	337	1	MS
20	Oleic acid	0.67	1.69	14.08	73, 75, 117, 129, 55, 67, 81, 96, 145, 217, 339	339	1	MS
					Sugars & sugar alcohols			
21	Glycerol	1.39	1.40	7.14	73, 147, 117, 103, 205, 133, 218, 299	191	3	MS
22	Erythritol	1.25	1.02	9.27	73, 147, 217, 103, 117, 205, 133, 189, 277, 307	277	4	MS
23	Xylose	0.64	0.91	10.54	73, 103, 147, 45, 75, 307, 133, 117, 189, 129, 59, 89	103	4	MS
24	Xylitol	1.32	1.37	10.97	73, 147, 217, 103, 129, 117, 205, 133, 45, 189, 319	217	5	MS
25	Fructose	1.33	1.58	12.09	73, 103, 217, 147, 133, 307, 45, 117, 89, 189	103	5	MS/STD
26	Glucose	1.28	1.03	12.33	73, 147, 205, 160, 103, 319, 217, 157, 117	103	5	MS/STD
27	myo-Inositol	2.29	1.94	13.51	73, 147, 217, 305, 191, 129, 133, 318, 103, 45, 204	305	6	MS/STD
28	Glyceryl-glycoside	0.09	1.69	14.70	73, 204, 147, 103, 205, 129, 217, 117, 133, 337, 169, 191	337	6	MS
29	Sucrose	0.22	1.22	16.58	73, 361, 147, 217, 103, 169, 129, 271, 191, 437, 319	157	8	MS
30	Lactose	0.78	0.85	17.17	73, 147, 204, 103, 217, 361, 129, 361, 319, 480	361	8	MS/STD
31	Maltose	1.81	1.58	17.49	73, 204, 147, 217, 103, 129, 361, 133, 117, 169, 319	204	8	MS/STD

[a] Tentatively identified metabolites were selected as differential variables based on *p*-value < 0.05 and VIP 1, 2 > 0.7; VIP 1 and VIP 2, value of variable importance in projection from PLS1 and PLS2 of PLS-DA model, respectively (Figure 3). [b] t_R, Retention time. [c] *m*/*z*, the selected ion for identification. [d] TMS, trimethylsilyl. [e] ID, identification. STD, standard compounds; MS, the mass spectra of NIST and in-house libraries.

Table S2. List of identified secondary metabolites selected as variables according to different cereal grains and hot peppers used in gochujang by using UHPLC-LTQ-IT-MS/MS data set.

No.	Tentative Metabolites [a]	t_R (min)	Measured Mass (*m*/*z*)	Adducts	MS^n Fragments (*m*/*z*) [b]	M.W.	λ_{max} (nm)	ID [c]
				Flavonoids				
32	Apigenin-*C*-hexoside-*C*-pentoside [d]	6.91	565	$[M + H]^+$	**565** > **547** > **529**, 511, 499, 469, 457, 451	564	200, 270	[1]
33	Luteolin-*O*-apiosyl-glucoside [f]	7.51	581	$[M + H]^+$	**581** > 449, **287**, 256 > 245	580	268, 287, 321, 366	[1,2]
34	Luteolin-*C*-hexoside [d,f]	7.68	449	$[M + H]^+$	**449** > 432, **287**	448	266, 283, 366, 414, 586	[1,2]
35	Genistin [f]	7.82	433	$[M + H]^+$	**433** > 415, **271** > 254, 243, 216, 154	432	200, 260	[3]
36	Apigenin-*O*-apiosyl-glucoside [e,f]	8.05	565	$[M + H]^+$	**565** > 433, **271** > 229, 154, 191	564	267, 335, 423	[4], Lib.
37	Quercetin-*O*-rhamnoside [d]	8.22	449	$[M + H]^+$	**449** > **303**, 287 > 285, 205, 165, 144	448	252, 286, 366	[1], STD
38	Apigenin-*O*-glucoside [f]	8.29	433	$[M + H]^+$	**433** > **271**, 154 > 253, 125	432	270	Lib., STD
39	Hydroxydaidzein [d,f]	8.97	271	$[M + H]^+$	**271** > **225** > 207,**197** > **141**,**129**, 113, 86	270	202, 269	[5], Lib.
40	Genistein-*O*-di-hexoside [d,e]	9.37	595	$[M + H]^+$	**595** > **433** > **289** > **271**, 253, 243, 225	594	204, 270	[3]
41	Daidzein [e,f]	9.46	255	$[M + H]^+$	**255** > 255, 237, 228	254	203, 268	[3]
42	Glycitein [e,f]	9.67	285	$[M + H]^+$	**285** > 267, 257, 240	284	202, 271	[3]
43	Luteolin [f]	9.78	287	$[M + H]^+$	**287** > 269, 229, 199	286	209, 345, 420	Lib.
44	Genistein [e,f]	10.64	271	$[M + H]^+$	**271** > 253, 215, 175, 153	270	289, 326, 366	[3]
				Soyasaponins				
45	Soyasaponin V [e,f]	11.36	959	$[M + H]^+$	**959** > 797, 635, **617** > **599**, 581, 441, 423	958	215	[6]
46	Soyasaponin I [e,f]	11.54	943	$[M + H]^+$	**943** > **797**, 781 > 635, 617, **599** > 581, 441, 423, 351	942	216	[6]
47	Soyasaponin II [e,f]	11.86	913	$[M + H]^+$	**913** > 895, 781, 767, 617, 605, 441	912	217	[6]
48	Soyasaponin III [e,f]	11.95	797	$[M + H]^+$	**797** > 635, 617, **599** > **581**, 441, 423 > 405, 353, 350, 201	796	217, 287	[6]
				Capsaicinoids & capsinoids				
49	Dihydrocapsiate [d,e,f]	9.99	309	$[M + H]^+$	**309** > **291** > 281, **263** > 236, 207	308	297, 321, 366	[7]
50	Nordihydrocapsaicin [e,f]	13.13	294	$[M + H]^+$	**294** > 170, **137** > 122, **109**, 91, 81 > 107, 94, 91, **81**	293	218, 280, 366, 377	[8]
51	Capsaicin [e,f]	13.29	306	$[M + H]^+$	**306** > **137**, 170, 182, 289 > 122, 109	305	218, 281, 366, 377	[8]
52	Dihydrocapsaicin [d,e,f]	14.03	308	$[M + H]^+$	**308** > 184, **137** > 122, 109, 91, 81	307	203, 218, 280	[8]
				Lipids				
53	Pinellic acid [d]	10.83	353	$[M + Na]^+$	**353** > 335 > 317, 289, 277, 261, 207, 167	330	213, 265, 334, 366	[9]
54	LysoPC 14:0 [d]	13.47	468	$[M + H]^+$	-	467	218	[10]
55	LysoPC 14:0 [d,f]	13.74	468	$[M + H]^+$	**468** > **450**, 184 > **391**, 268, 255, 181, 163	467	218	[10]
56	LysoPC 18:3 [d,f]	13.89	518	$[M + H]^+$	**518** > **500**, 184 > **441**, 305, 288, 182, 160	517	219	[10]
57	LysoPE 18:2 [d,f]	14.20	478	$[M + H]^+$	**478** > 460, **337** > 319, **263**, 245, 189, 175, 133, 121, 109	477	219	[10]
58	LysoPE 18:2 [d,f]	14.46	478	$[M + H]^+$	**478** > **460**, 337 > 417, 306, **263**, 155 > **245**, 165, 149, 137	477	219, 376	[10]
59	LysoPC 18:2 [d,f]	14.58	520	$[M + H]^+$	**520** > **502**, 184 > 443	519	220	[11]
60	LysoPC 18:2 [d,f]	14.87	520	$[M + H]^+$	**520** > **502**, 184 > 443	519	219, 278	[11]
61	LysoPE 16:0 [e,f]	15.14	454	$[M + H]^+$	**454** > **436**, 313 > 418, **393**, 282 > 239, **155**	453	220	[10]
62	LysoPC 16:0 [d,f]	15.41	496	$[M + H]^+$	**496** > **478**, 184 > 419	495	220	[11]
63	LysoPC 16:0 [d,e,f]	15.79	496	$[M + H]^+$	**496** > 478, 184 > 419	495	220	[11]
64	LysoPC 18:1 [d,f]	15.95	522	$[M + H]^+$	-	521	220	[11]
65	LysoPC 18:1 [d,f]	16.35	522	$[M + H]^+$	**522** > **504**,184 > 445, 163	521	220	[11]
66	Linoleic ethanolamide [d]	17.15	324	$[M + H]^+$	**324** > 307, 263, 245 > 289, 245, 175, 109	323	221	[12]

[a] Tentative metabolites were identified by comparing retention time (t_R), fragment pattern (MS^n), molecular weight (M.W.), and λ_{max} (nm) with reference. [b] Numbers in bold mean major peak in the MS spectrum. [c] ID, Identification; STD, standard compounds; Ref., reference; Lib., in-house libraries. [d] Selected metabolites as variables according to different cereal grains in PLS-DA score plot (Figure 3B). [e] Significantly different metabolites between RbG-CA and RbG-CAY were selected based on *p*-value < 0.05 and VIP > 1 derived from OPLS-DA score plot (Figure 4). [f] Significantly different metabolites between RwG-CA and RwG-CF were selected based on *p*-value < 0.05 and VIP > 1 derived from OPLS-DA score plot (Figure 4).

Table S3. Relative contents of significantly different metabolites according to different cereal grains used in commercial gochujang.

No.	Tentative Metabolites [#]	Relative contents ** (Mean ± SD × 10^4)		
		WG	RbG	RwG
	Amino acids			
1	Leucine	5.23 ± 2.32 [a]	2.29 ± 0.39 [b]	3.34 ± 1.20 [c]
2	Valine	1.60 ± 0.85 [a]	0.77 ± 0.18 [b]	1.08 ± 0.39 [b]
3	Isoleucine	7.22 ± 4.03 [a]	3.32 ± 1.12 [b]	4.32 ± 1.65 [b]
4	Proline	32.21 ± 14.1 [a]	19.89 ± 6.39 [b]	17.38 ± 5.07 [b]
5	Glycine	14.69 ± 6.45 [a]	5.76 ± 0.83 [b]	8.92 ± 3.08 [c]
6	Serine	5.63 ± 2.79 [a]	1.99 ± 0.5 4 [b]	2.75 ± 1.18 [b]
7	Threonine	1.42 ± 0.77 [a]	0.72 ± 0.27 [b]	0.85 ± 0.34 [b]
8	Aspartic acid	1.12 ± 0.22 [a]	0.75 ± 0.18 [b]	1.10 ± 0.43 [a]
9	Pyroglutamic acid	59.17 ± 10.5 [a]	24.78 ± 6.20 [b]	19.83 ± 8.65 [b]
10	GABA	3.70 ± 0.79 [a]	4.26 ± 0.22 [a]	5.12 ± 1.53 [b]
11	Glutamic acid	8.39 ± 4.81 [a]	4.03 ± 1.81 [b]	2.84 ± 2.43 [b]
12	Phenylalanine	4.53 ± 2.64 [a]	1.51 ± 0.70 [b]	2.17 ± 1.02 [b]
	Organic acids			
13	Propanoic acid	0.86 ± 0.07 [a]	0.99 ± 0.18 [b]	0.74 ± 0.09 [c]
14	Fumaric acid	0.21 ± 0.05 [a]	0.18 ± 0.02 [b]	0.12 ± 0.02 [c]
15	Malic acid	2.24 ± 0.52 [a]	1.71 ± 0.26 [b]	1.37 ± 0.23 [c]
16	Citric acid	41.88 ± 10.7 [a]	31.82 ± 6.98 [b]	29.50 ± 10.8 [b]
17	Gluconic acid	2.20 ± 1.90 [a]	0.55 ± 0.15 [b]	1.55 ± 0.75 [a]
	Fatty acids			
18	Palmitic acid	13.18 ± 5.39 [a]	11.95 ± 2.80 [a]	19.17 ± 8.54 [b]
19	Linoleic acid	0.12 ± 0.06 [a]	0.14 ± 0.08 [a]	0.08 ± 0.05 [b]
20	Oleic acid	0.04 ± 0.02 [a]	0.07 ± 0.03 [b]	0.03 ± 0.02 [a]
	Sugars & sugar alcohols			
21	Glycerol	14.76 ± 0.56 [a]	14.74 ± 0.74 [a]	13.65 ± 1.05 [b]
22	Erythritol	0.30 ± 0.20 [a]	0.24 ± 0.11 [ab]	0.16 ± 0.09 [b]
23	Xylose	8.21 ± 1.96 [a]	5.46 ± 1.00 [b]	6.57 ± 3.23 [ab]
24	Xylitol	31.83 ± 14.0 [a]	32.04 ± 8.57 [a]	19.46 ± 9.90 [b]
25	Fructose	243.81 ± 16.5 [a]	250.62 ± 6.73 [a]	219.28 ± 26.0 [b]
26	Glucose	66.86 ± 2.82 [a]	70.40 ± 1.65 [ab]	74.69 ± 9.45 [b]
27	myo-Inositol	7.00 ± 1.07 [a]	6.12 ± 0.78 [b]	4.54 ± 0.77 [c]
28	Glyceryl-glycoside	1.47 ± 0.79 [a]	0.44 ± 0.26 [b]	1.30 ± 0.54 [a]
29	Sucrose	0.20 ± 0.24 [a]	1.10 ± 1.70 [b]	0.14 ± 0.11 [a]
30	Lactose	42.46 ± 4.52 [a]	45.76 ± 2.77 [b]	44.90 ± 2.84 [b]
31	Maltose	1.91 ± 0.76 [a]	2.70 ± 0.58 [a]	4.74 ± 2.08 [b]
	Flavonoids			
32	Apigenin-*C*-hexoside-*C*-pentoside	1.23 ± 0.91 [a]	0.20 ± 0.77 [b]	0.40 ± 0.89 [b]
34	Luteolin-*C*-hexoside	2.21 ± 1.71 [a]	3.70 ± 1.81 [b]	2.85 ± 1.30 [ab]
37	Quercetin-*O*-rhamnoside	3.71 ± 1.87 [a]	5.18 ± 1.69 [b]	3.29 ± 1.40 [a]
39	Hydroxydaidzein	1.53 ± 0.55 [a]	0.16 ± 0.25 [b]	0.86 ± 1.10 [c]
40	Genistein-*O*-di-hexoside	1.70 ± 1.58 [a]	3.20 ± 2.11 [a]	8.19 ± 5.15 [b]

Capsaicinoids & capsinoids				
49	Dihydrocapsiate	43.66 ± 58.7 [a]	11.60 ± 5.00 [b]	14.19 ± 12.2 [b]
52	Dihydrocapsaicin	109.28 ±17.2[a]	136.62 ± 18.5 [b]	150.82 ± 47.4 [b]
lipids				
53	Pinellic acid	3.18 ±1.04 [a]	3.24 ± 0.42 [a]	3.72 ± 0.48 [b]
54	LysoPC 14:0	0.08 ± 0.14 [a]	1.11 ± 0.47 [b]	0.25 ± 0.23 [a]
55	LysoPC 14:0	1.29 ± 1.83[a]	14.84 ± 6.28 [b]	3.27 ± 3.29 [a]
56	LysoPC 18:3	2.13 ± 0.99 [a]	2.53 ± 0.67 [a]	0.63 ± 0.43 [b]
57	LysoPE 18:2	3.94 ±4.33[a]	2.20 ± 0.31[b]	1.00 ± 0.56 [b]
58	LysoPE 18:2	5.85 ± 3.42 [a]	13.02 ± 2.56 [b]	3.86 ± 2.92 [a]
59	LysoPC 18:2	10.65 ± 10.2 [a]	7.08 ± 1.51 [b]	2.47 ± 1.50 [c]
60	LysoPC 18:2	41.41 ± 28.8 [a]	70.30 ±16.2 [b]	19.47 ± 15.6 [c]
62	LysoPC 16:0	4.23 ± 3.34 [a]	10.61 ± 3.89 [b]	4.20 ± 3.01 [a]
63	LysoPC 16:0	33.99 ± 32.7 [a]	91.76 ± 27.9 [b]	34.57 ± 31.0 [a]
64	LysoPC 18:1	2.55 ± 1.76 [a]	2.08 ± 0.74 [a]	1.09 ± 0.61 [b]
65	LysoPC 18:1	8.07 ± 7.12 [a]	16.17 ± 6.54 [b]	5.89 ±5.98 [a]
66	Linoleic ethanolamide	66.22 ± 14.5 [a]	13.13 ± 3.80 [b]	24.87 ± 17.7 [c]

[a,b,c] Different letters indicate significant differences calculated by Duncan's multiple comparison test (p-value < 0.05). [#] Tentative identified metabolites were selected as variables based on variable importance in projection (VIP > 0.7) from PLS-DA and p-value < 0.05. [**] Relative contents; values are means of relative peak areas in triplicate from alignment data ± standard deviation (SD); WG, wheat gochujang; RbG, brown rice gochujang; RwG, white rice gochujang.

References

1. Wahyuni, Y.; Ballester, A.R.; Sudarmonowati, E.; Bino, R.J.; Bovy, A.G. Metabolite biodiversity in pepper (*Capsicum*) fruits of thirty-two diverse accessions: Variation in health-related compounds and implications for breeding. *Phytochem.* **2011**, *72*, 1358–1370.
2. Jeong, W.Y.; Jin, J.S.; Cho, Y.A.; Lee, J.H.; Park, S.; Jeong, S.W.; Kim, Y.H.; Lim, C.S.; Abd El-Aty, A.M.; Kim, G.S.; et al. Determination of polyphenols in three *Capsicum annuum* L. (bell pepper) varieties using high-performance liquid chromatography-tandem mass spectrometry: Their contribution to overall antioxidant and anticancer activity. *J. Sep. Sci.* **2011**, *34*, 2967–2974.
3. Cavaliere, C.; Cucci, F.; Foglia, P.; Guarino, C.; Samperi, R.; Laganà, A. Flavonoid profile in soybeans by high-performance liquid chromatography/tandem mass spectrometry. *Rapid Commun. Mass Spectrom.* **2007**, *21*, 2177–2187.
4. Li, P.; Jia, J.; Zhang, D.; Xie, J.; Xu, X.; Wei, D. In vitro and in vivo antioxidant activities of a flavonoid isolated from celery (*Apium graveolens* L. var. dulce). *Food Funct.* **2014**, *5*, 50–56.
5. Lee, S.; Seo, M.H.; Oh, D.K.; Lee, C.H. Targeted metabolomics for *Aspergillus oryzae*-mediated biotransformation of soybean isoflavones, showing variations in primary metabolites. *Biosci. Biotechnol. Biochem.* **2014**, *78*, 167–174.
6. Lee, S.Y.; Lee, S.; Lee, S.; Oh, J.Y.; Jeon, E.J.; Ryu, H.S.; Lee, C.H. Primary and secondary metabolite profiling of doenjang, a fermented soybean paste during industrial processing. *Food Chem.* **2014**, *165*, 157–166.
7. Singh, S.; Jarret, R.; Russo, V.; Majetich, G.; Shimkus, J.; Bushway, R.; Perkins, B. Determination of capsinoids by HPLC-DAD in *Capsicum* species. *J. Agric. Food Chem.* **2009**, *57*, 3452–3457.
8. Choi, S.H.; Suh, B.S.; Kozukue, E.; Kozukue, N.; Levin, C.E.; Friedman, M. Analysis of the contents of pungent compounds in fresh Korean red peppers and in pepper-containing foods. *J. Agric. Food Chem.* **2006**, *54*, 9024–9031.
9. Kim, G.R.; Jung, E.S.; Lee, S.; Lim, S.H.; Ha, S.H.; Lee, C.H. Combined mass spectrometry-based metabolite profiling of different pigmented rice (*Oryza sativa* L.) seeds and correlation with antioxidant activities. *Molecules* **2014**, *19*, 15673–15686.
10. Fang, N.; Yu, S.; Badger, T.M. LC-MS/MS analysis of lysophospholipids associated with soy protein isolate. *J. Agric. Food Chem.* **2003**, *51*, 6676–6682.

11. Zaima, N.; Yoshimura, Y.; Kawamura, Y.; Moriyama, T. Distribution of lysophosphatidylcholine in the endosperm of *Oryza sativa* rice. *Rapid Commun. Mass Spectrom.* **2014**, *28*, 1515–1520.
12. Palandra, J.; Prusakiewicz, J.; Ozer, J.S.; Zhang, Y.; Heath, T.G. Endogenous ethanolamide analysis in human plasma using HPLC tandem MS with electrospray ionization. *J. Chromatogr. B* **2009**, *877*, 2052–2060.
